# Cost-effectiveness of preventative therapies for postmenopausal women with osteopenia

**DOI:** 10.1186/1472-6874-7-6

**Published:** 2007-04-17

**Authors:** Eric S Meadows, Robert Klein, Matthew D Rousculp, Lee Smolen, Robert L Ohsfeldt, Joseph A Johnston

**Affiliations:** 1 Eli Lilly and Company, Indianapolis, IN, USA; 2 Medical Decision Modeling Inc., Indianapolis, IN, USA; 3 Texas A&M Health Science Center, College Station, TX, USA; 4 MedImmune, Gaithersburg, MD, USA

## Abstract

**Background:**

Limited data are available regarding the cost-effectiveness of preventative therapies for postmenopausal women with osteopenia. The objective of the present study was to evaluate the cost-effectiveness of raloxifene, alendronate and conservative care in this population.

**Methods:**

We developed a microsimulation model to assess the incremental cost and effectiveness of raloxifene and alendronate relative to conservative care. We assumed a societal perspective and a lifetime time horizon. We examined clinical scenarios involving postmenopausal women from 55 to 75 years of age with bone mineral density T-scores ranging from -1.0 to -2.4. Modeled health events included vertebral and nonvertebral fractures, invasive breast cancer, and venous thromboembolism (VTE). Raloxifene and alendronate were assumed to reduce the incidence of vertebral but not nonvertebral fractures; raloxifene was assumed to decrease the incidence of breast cancer and increase the incidence of VTEs. Cost-effectiveness is reported in $/QALYs gained.

**Results:**

For women 55 to 60 years of age with a T-score of -1.8, raloxifene cost approximately $50,000/QALY gained relative to conservative care. Raloxifene was less cost-effective for women 65 and older. At all ages, alendronate was both more expensive and less effective than raloxifene. In most clinical scenarios, raloxifene conferred a greater benefit (in QALYs) from prevention of invasive breast cancer than from fracture prevention. Results were most sensitive to the population's underlying risk of fracture and breast cancer, assumed efficacy and costs of treatment, and the discount rate.

**Conclusion:**

For 55 and 60 year old women with osteopenia, treatment with raloxifene compares favorably to interventions accepted as cost-effective.

## Background

Osteoporosis has been operationally defined as a bone mineral density (BMD) at least 2.5 standard deviations below that seen in young healthy women (T-score < -2.5) and osteopenia as a BMD T-score between -1 and -2.5[1]. Although the risk of fracture is greater in postmenopausal women with osteoporosis than with osteopenia, there are far more postmenopausal women with BMD values in the normal or osteopenic range[2]. As a result, more fractures occur in women with osteopenia than with osteoporosis [3-6]. Guidelines generally agree that women with osteoporosis should receive pharmacotherapy [7-9] for fracture prevention but consensus has not been reached for the management of women with osteopenia. With up to 50% of postmenopausal women in the US estimated to have osteopenia,[2] the optimal approach to fracture prevention in these women is a question of vital public health interest.

Conservative care for osteopenic women includes weight-bearing exercise and calcium/vitamin D supplementation. Pharmacological treatment options include bisphosphonates and raloxifene. The effects of bisphosphonates are largely limited to bone tissue due to their long-term incorporation into the bone matrix[10]. The selective estrogen receptor modulator (SERM) raloxifene is indicated for the prevention and treatment of osteoporosis and is also associated with multiple extra-skeletal effects[11]. Raloxifene differs from bisphosphonates and from other SERMs in that the results of large clinical trials have demonstrated that it reduces the incidence of both vertebral fractures and breast cancer [12-14]. An important adverse event associated with raloxifene is an increased incidence of venous thromboembolism[15,16]. Selecting a bisphosphonate, raloxifene, or conservative management for women with osteopenia requires consideration of the benefits, risks, and costs of each option.

A recent study concluded that alendronate, a commonly prescribed bisphosphonate, was not cost-effective relative to conservative care for osteopenic women in the absence of additional risk factors for fracture[17]. The effectiveness and cost-effectiveness of raloxifene in this population have yet to be established. To that end, we developed a decision analytic model to examine the effectiveness and cost-effectiveness of raloxifene and alendronate relative to conservative care for postmenopausal women with low bone mass who have not yet experienced a fracture.

## Methods

We developed a microsimulation model to assess cost-effectiveness from a societal perspective. The structure of the model and additional methodological details are included in an additional file [see Additional File 1]. We examined clinical scenarios including postmenopausal women without any pre-existing fractures, ranging in starting age from 55 to 75 with BMD T-scores ranging from -1.0 to -2.4 at the femoral neck. For each age and T-score combination, a patient's risk of breast cancer was assumed in the base case to be equal to the population mean and varied in sensitivity analyses. Three management strategies, 5 years of raloxifene, 5 years of alendronate, or no drug therapy, were considered. Patients were assumed to be 100% compliant with therapy but this assumption was varied in sensitivity analyses. All patients were assumed to be receiving supplemental calcium and vitamin D. In each cycle a patient may die or incur any one or more of four fragility fracture types (hip, vertebral, wrist, and other), breast cancer, and/or a venous thromboembolism (VTE). Cohorts of 100,000 women were simulated until age 100 or death. Age-dependent mortality was modeled using 2002 data[18]. Health states were assigned utilities, reflecting patient preferences, from the literature and effectiveness was assessed in quality-adjusted life years (QALYs). Cost-effectiveness was assessed in terms of dollars per event avoided and dollars per QALY. Costs and outcomes were discounted at a 3% annual rate in the base case. Key parameters and assumptions used in the model are summarized in Table 1 and additional information is available in the technical appendix.

**Table 1:** Key model parameters

**Parameter**	**Value**
**Direct medical costs, $**	
Raloxifene, annual cost	982 [34]
Alendronate, annual cost	880 [34]
Hip Fracture, First year	30,499
Direct Medical Costs	19,566 [36]
Long Term Care, year 1	10,933 [39]
Hip Fracture, Subsequent Years	7,723 [36]
Vertebral Fracture	8,002 [36]
Vertebral Fracture, Non-Clinical	0
Other Fracture	6,289 [36]
Wrist Fracture	4,344 [36]
Breast Cancer	
Stage I, years 1–4	23,290; 7,763; 3,882; 1,086 [38]
Stage II, years 1–4	24,066; 6,287; 6,987; 7,375 [38]
Stage III, years 1–3	41,922; 47,357; 3,882 [38]
Stage IV, years 1–3	57,448; 17,079; 2,329 [38]
Fatal VTE	6,665 [37]
Non-Fatal VTE	17,034 [35]
**Relative event risks with treatment**	
Vertebral fracture, alendronate	0.54 [25]
Vertebral fracture, raloxifene	0.53 [23]
Invasive breast cancer, raloxifene, years 2–5	0.28 [30]
VTE, raloxifene, years 1 and 2	6.2 [15]
**Health state utilities**	
Initial utility	0.84 [55]
Relative utilities	
Post fracture	
Hip, year 1, years 2+	0.792, 0.813 [56]
Vertebral, year 1, years 2+	0.69, 0.905 [56]
Non-clinical vertebral, years 1–6	0.905 [57]
Other, year 1, years 2+	0.896, 0.968 [56]
Wrist, year 1, years 2+	0.976, .999 [56]
Breast cancer	
Stage I, year 1, year 2–5, year 6+	0.85, 0.91, 0.99 [58-61]
Stage II, year 1, years 2+	0.72, 0.87 [58-61]
Stage III, year 1, years 2+	0.62, 0.84 [58-61]
Stage IV, year 1, year 2, 3+	0.42, 0.64, 0.84 [58-61]
Terminal	0.23 [59]
Post VTE, year 1, years 2+	0.9, 0.986 [44]

Abbreviations: VTE, venous thromboembolism.

### Event incidence rates

Age and T-score dependent incidences for spine (both radiographic and clinical), hip, wrist and other fractures were calculated from published data[19]. We assumed that 35% of spine fractures would be clinically apparent[20]. Post-fracture risk multipliers were used to calculate increased rates for subsequent fractures[21]. Following a hip fracture, 23% of the observed increase in mortality at age 60 was assumed to be attributable to the hip fracture event[22]. Fracture reduction efficacy for subgroups of women with T-scores in the osteopenic range have been reported for both raloxifene[23] and alendronate[24]. While there is disagreement about whether alendronate reduces risk of vertebral fracture in osteopenic women [24-27], we assumed that the vertebral fracture risk reduction with alendronate that was reported for women with osteoporosis[25] also applied to women with osteopenia. Neither therapy has demonstrated efficacy in preventing nonvertebral fractures in osteopenic women [23-25]. After treatment cessation, residual fracture reduction benefits were phased out linearly over five years.

Five-year risks of invasive breast cancer were obtained from the Surveillance, Epidemiology, and End Results database[28]. Stage at diagnosis was calculated by pooling 1996–2000 with 2001–2002 data available from the Centers for Disease Control[29]. Age-dependent breast cancer mortalities were computed by stage at diagnosis. The effect of raloxifene on reducing the incidence of invasive breast cancer was assumed to begin in the second year[30] and phase out over five years after raloxifene treatment ends. Alendronate was assumed not to affect the risk of breast cancer.

The incidence of VTE in 67 year old women eligible for and receiving raloxifene treatment was obtained from the placebo group of the MORE trial[15] and extrapolated to other ages[31]. Recurrence rates for VTE were 8%[32] in the first year and 2% in all subsequent years[33]. We assumed 5% of VTEs were fatal[32]. Raloxifene was assumed to confer a 6.2-fold increase in VTE incidence during the first 2 years of raloxifene treatment, with a return to baseline beginning in the 3^rd ^year of therapy[15,16]. If a patient who received raloxifene experienced a VTE or developed breast cancer, treatment was immediately stopped.

### Costs

Medication costs for alendronate and raloxifene reflect April 2006 Net Wholesale Price (NWP)[34]. Other cost estimates were obtained from published literature [35-39] and inflated to April 2006 US dollars using the healthcare component of the Consumer Price Index.(Table 1).

### Sensitivity Analyses

Alternative scenarios were considered to understand the importance of the duration of therapy and the patient population's risk of fracture, breast cancer, and VTE. One-way sensitivity analyses were used to determine the influence of other model parameters. In probabilistic sensitivity analyses (presented in the technical appendix), input values were sampled from lognormal distributions for event costs and generalized beta distributions for health state utilities and the relative risk of events with treatment. Using these distributions, 1000 samples of 1000 simulated patients were analyzed.

## Results

### Base case

For 60 year old women with a T-score in the middle of the osteopenic range (-1.8) and with the population mean risk of breast cancer and VTE, 5 years of treatment with raloxifene has an incremental lifetime discounted cost of $3,726 per patient. The expected net total decrease in clinical vertebral fractures (22.4 fewer cases per 1,000 women) and breast cancers (23.1 fewer cases per 1,000 women) and increase in VTEs (12.1 more cases per 1,000 women) results in a lifetime discounted cost of $111,257 per event avoided. The incremental cost-effectiveness ratio for 60 year old women in the middle of the osteopenic range (T-score = -1.8) is approximately $50,000 per quality-adjusted life year (QALY) relative to conservative care.(Table 2) Similar results were obtained for 55 year old women. Raloxifene was less cost-effective for women starting therapy at age 65 and the cost-effectiveness ratios increased further for 70 and 75 year old women.

**Table 2:** Incremental cost-effectiveness ratios (ICERs) for 5 years of drug therapy or no therapy, T-score = -1.8.

		**Cost ($)**		**Effectiveness (QALY)**		ICER* ($/QALY)
			
**Age**	**Therapy**	**Total**	**Δ**	**Total**	**Δ**	
55	Conservative care	20,342	-	14.270	-	-
	Raloxifene	24,169	3,827	14.351	0.081	47,247
	Alendronate	24,370	201	14.303	-0.048	Dominated
60	Conservative care	16,773	-	12.747	-	
	Raloxifene	20,499	3,726	12.823	0.076	49,026
	Alendronate	20,748	249	12.782	-0.041	dominated
65	Conservative care	13,541	-	11.131	-	-
	Raloxifene	17,240	3,699	11.196	0.065	56,908
	Alendronate	17,471	231	11.166	-0.030	dominated
70	Conservative care	10,643	-	9.474	-	
	Raloxifene	14,355	3,712	9.528	0.054	68,741
	Alendronate	14,513	158	9.505	-0.023	dominated
75	Conservative care	8,084	-	7.808	-	-
	Alendronate	11,860	3,776	7.833	0.025	dominated*
	Raloxifene	11,861	3,777	7.845	0.037	102,081

The ICERs were calculated using additional significant figures in the incremental cost and QALYs. Abbreviations: QALY, quality-adjusted life year; ICER, incremental cost-effectiveness ratio

• **dominated by extended dominance (raloxifene has greater effectiveness and a lower cost-effectiveness ratio)**

Under base case assumptions, alendronate therapy was more expensive and less effective than raloxifene for all combinations of age (55 to 75) and T-score (-1.0 to -2.4) considered. In situations where raloxifene therapy might not be considered a viable treatment option, such as in patients with a history of VTE or with active neoplasms, alendronate costs approximately $113,000/QALY compared to conservative care for 60 year old women with a T-score of -1.8.

**Figure 1:**
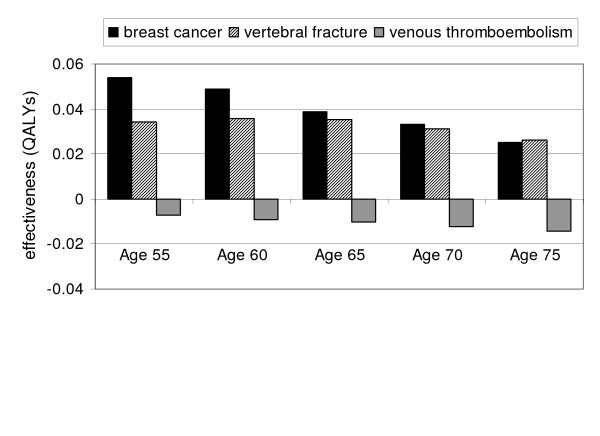
**Relative contributions of the skeletal and extraskeletal effects of raloxifene**. The total expected effectiveness is the sum of the contributions from the reduced incidence of vertebral fractures and breast cancers minus the QALYs lost from the increased incidence of venous thromboembolism. Abbreviations: QALY, quality-adjusted life year.

The influence of the extra-skeletal effects with raloxifene is summarized in Figure 1. From age 55 to 70, the increase in quality-adjusted life expectancy from the prevention of invasive breast cancers is greater than for fracture prevention. At age 75, the increased risk of VTEs with raloxifene offsets nearly 30% of the effects from fracture and breast cancer prevention.

### Importance of Patient Population Characteristics

**Figure 2:**
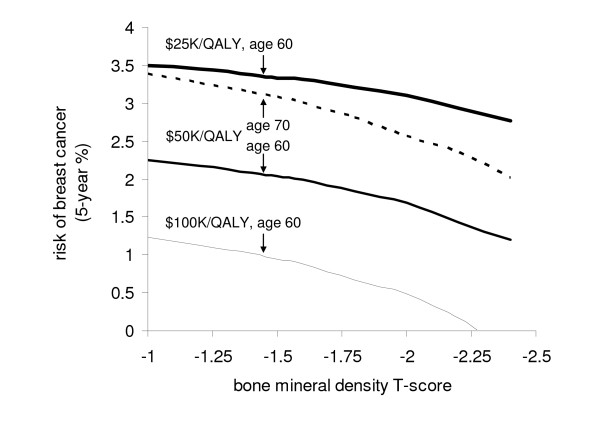
**Cost-effectiveness thresholds for raloxifene treatment of postmenopausal women at varying ages, T-score, and risk of breast cancer**. Patient populations with a T-score worse or a breast cancer risk greater than that shown at each line would be considered cost-effective at the indicated societal willingess-to-pay. The influence of age is demonstrated by showing the $50,000/QALY threshold at 60 and 70 years of age.

The effect of T-score and risk of breast cancer on raloxifene's cost-effectivness ratio is summarized in Figure 2. At each age, raloxifene was more cost-effective at lower T-scores and higher risk of breast cancer. For women near the threshold of osteoporosis (T-score = -2.4), the incremental cost-effectiveness ratio with raloxifene improved to $36,972, while for women with a T-score of -1.0, it increased to $63,027. For 60 year old women, the cost-effectiveness ratio for raloxifene was less than $50,000/QALY for all osteopenic women with a 5-year risk of breast cancer of greater than 2.25%. For 60 year old women with a 5-year risk of breast cancer of 1.87%, which is the age-dependent population mean risk, the cost-effectiveness ratio for raloxifene was less than $50,000/QALY when the T-score was -1.8 or worse. Raloxifene was cost-saving in 60 year old women with over 4 times the population relative risk of breast cancer (e.g., a woman who has received 2 or more breast biopsies, at least 1 of which resulted in a diagnosis of atypical hyperplasia, and 1 first degree relative with a history of breast cancer) at a T-score of -1.8.

### Sensitivity Analyses

If the duration of raloxifene therapy was shortened to 2 years or extended to 7 years, the cost effectiveness ratio increased to $71,442 or $50,944 respectively. Including mortality attributable to vertebral fractures improved the cost-effectiveness ratio to $45,721. Reducing compliance with therapy to 80% or 50% increased the cost-effectiveness ratio to $50,660 or $58,260. Although never shown to do so in a non-osteoporotic population, if alendronate was assumed to reduce the incidence of nonvertebral fractures and the risk of breast cancer was equal to the population mean, then alendronate was found to be more cost-effective than raloxifene in women over the age of 60 with a T-score of -1.9 or lower. If the price of alendronate was reduced by over 50% to $1/day, then the cost-effectiveness ratio compared to conservative care was $46,714 for 60 year old women with a T-score of -1.8. For a SERM with similar efficacy as raloxifene in preventing vertebral fractures but with no effect on breast cancer or VTEs, the cost per QALY was $123,262.

**Figure 3:**
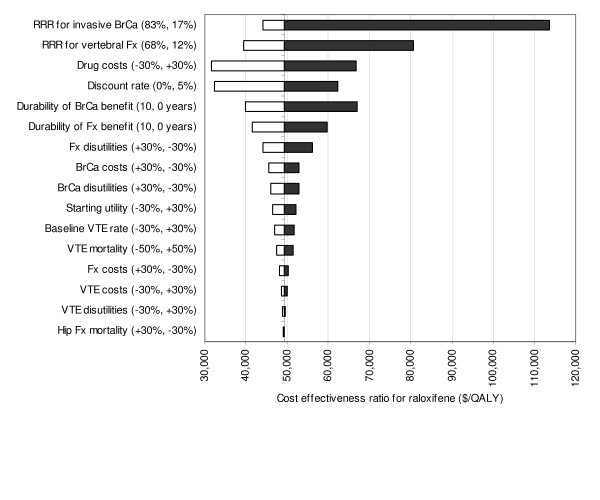
**Univariate sensitivity analyses for raloxifene**. The black bars indicate increases and white bars indicate decreases in the incremental cost-effectiveness ratio. The values shown in parentheses correspond to the range of input values. Abbreviations: RRR, relative risk reduction; BrCa, breast cancer; Fx, fracture; VTE, venous thromboembolism.

Additional sensitivity analyses are summarized in Figure 3. The results were sensitive to the discount rate and therapy parameters including the efficacy magnitude, efficacy duration after treatment cessation, and medication costs. Patient preferences for post-vertebral fracture and breast cancer states were of moderate importance. The results were less sensitive to the attributable mortality following hip fracture, fracture costs, or any of the VTE parameters.

## Discussion

Antiresorptive agents are considered effective in reducing the incidence of fracture and have favorable cost-effectiveness profiles for most postmenopausal women with osteoporosis[40]. Whether these mediations should be used in patients with osteopenia is much less clear even though therapy preserves bone mass and bone structure in these women [7-9]. Because up to 50% of postmenopausal women have osteopenia[2], and more than half of fragility fractures occur in these women[3], this issue is particularly relevant from a health policy perspective. A recent study found that alendronate would likely not be considered cost-effective for the treatment of women with osteopenia diagnosed on the basis of BMD alone[17]. If only fracture prevention is considered, alternative treatment options such as raloxifene, calcitonin, or other bisphosphonates would be expected to have similarly unfavorable cost-effectiveness profiles in women with osteopenia. However, previous decision analyses have found that the extraskeletal effects of raloxifene are important considerations[21,41-45]. In the current study, assuming a societal willingness-to-pay of $50,000 per QALY, raloxifene would be considered cost-effective for 55 and 60 year old women with T-scores in the lower half of the osteopenic range. For 60 year old women with a 5-year risk of breast cancer that is 50% greater than the population average, raloxifene treatment would cost less than $50,000/QALY for women throughout the osteopenic range (T-score between -1 and -2.5). Examples of additional breast cancer risk factors that would increase the risk of 60 year old women more than 50% include having at least 1 breast biopsy with atypical hyperplasia or at least 1 first degree relative with a history of breast cancer[44]. The extraskeletal effects of raloxifene contributed substantially to its effectiveness, with the reduction in the incidence of breast cancers contributing more to the gain in QALYs compared to fracture prevention for women under the age of 75.

The results for both alendronate and raloxifene were sensitive to assumptions about cost and efficacy. Generic alendronate is currently expected to become available in 2008 and if the price of alendronate drops to $1/day, the cost-effectiveness ratio would be similar to that of raloxifene for 60 year old women with a T-score of -1.8. Consistent with a prior study[17], our findings suggest that at its current price alendronate would not be considered cost-effective for osteopenic women unless it was assumed to decrease the incidence of nonvertebral fractures and restricted to use in women with bone mineral densities nearly in the osteoporotic range.

Previous studies[21,41,43-45,47] have indicated that the cost-effectiveness of raloxifene, which as of 2006 is indicated only for the prevention and treatment of osteoporosis, was driven largely by its associated extraskeletal effects. Although most previous work has focused on patients with osteoporosis[21,43-45], they have nevertheless demonstrated that the age, risk of fracture, and risk of breast cancer of the target population are key considerations. For example, a model of raloxifene versus placebo found that for 60 year-old women approximately 80% and 20% of overall QALYs gained from raloxifene were attributable to breast cancer prevention and vertebral fracture prevention, respectively, even for an osteoporotic patient population[44]. Another study that modeled healthy postmenopausal women found that raloxifene would be considered a cost-effective alternative to hormone replacement therapy (HRT)[41]. A decision analysis that considered raloxifene, alendronate, or hormone replacement therapy, gains in life expectancy were dependent on a woman's individual risk profile for osteoporosis, breast cancer, and cardiovascular disease[42]. However, the latter two studies were completed prior to the findings from the Women's Health Initiative (WHI) that demonstrated net harm with HRT in relatively healthy postmenopausal women[48]. A comparative economic analysis in a preventative setting that included the WHI results found that raloxifene was more cost-effective than alendronate, while hormone replacement therapy would be predicted to result in net harm[47]. The current study is the first that has incrementally compared raloxifene and alendronate treatment in osteopenic women.

### Limitations

First, as with any simulation, the actual clinical situation was simplified to avoid creating an overly complex model. For example, common side effects such as gastrointenstinal upset for bisphosphonates and hot flashes or leg cramps for raloxifene have not been considered. Second, the usefulness of the results depends on the quality of the model inputs and there was substantial uncertainty in several of the parameters. We have addressed this issue through sensitivity analyses but the results of these analyses span a considerable range for some parameters. Third, although the results were dependent on the patient population's risk of developing invasive breast cancer, available methods to calculate the patient's 5-year risk, such as the Gail model[46], are not in widespread use and do not discriminate which individual patients will develop breast cancer[49]. However, for 60 year old women with T-scores in the lower half of the osteopenic range, even when they do not have an elevated risk of breast cancer for their age, raloxifene compared favorably with the commonly used benchmark of $50,000/QALY for a therapy to be considered cost-effective. Finally, the results apply only to postmenopausal women with osteopenia who are otherwise relatively healthy. A recently completed large clinical trial demonstrated that, in women with coronary heart disease (CHD) or an increased risk of CHD, raloxifene was not associated with an increase or decrease in the incidence of coronary events or stroke but there was an increase in stroke mortality[50]. Osteonecrosis of the jaw (ONJ) is a rare but serious adverse event associated with bisphosphonate use[51]. Most, although not all, cases of ONJ have been reported in patients with multiple myeloma or metastatic cancer[51]. We have not included stroke or ONJ in the current model because women at an increased risk for CHD or with cancer would be considered outside the scope of the current study.

These results are not necessarily applicable to other SERMs with different efficacy and safety profiles. For example, tamoxifen has proven efficacy for reducing the incidence of primary and recurrent breast cancer but is also associated with an increased risk of endometrial cancer[52]. In the primary prevention setting, tamoxifen is most cost-effective for younger women at increased risk of breast cancer who have had a hysterectomy[52]. As new SERMs are developed, the specific balance of skeletal (i.e., vertebral and nonvertebral risk reduction) and extraskeletal effects will require new analyses in order to understand the most favorable patient population for each molecule.

An algorithm from the World Health Organization to derive the absolute risk of fracture[54] based on a number of clinical inputs is expected to be released in 2007. The results of the current study, based on T-scores, should be re-calibrated to consider a patient's absolute risk of fracture to be comparable with future cost-effectiveness studies. However, advanced age is one of the most important risk factors for fracture and was one of the variables examined here.

## Conclusion

While the present study suggests that the use of raloxifene to prevent fractures in postmenopausal women with osteopenia may be cost-effective on a population level, the optimal treatment decision for an individual patient will depend upon her unique profile of risks for various positive and negative outcomes and values attributed to potential health states, including that of taking a treatment with evident side effects and largely unobservable benefits. As our ability to estimate patient-specific disease risks improves, the importance of integrating these risk assessments and individualizing complex preventative treatment decisions will grow.

## Competing interests

Funding for the study was provided by Eli Lilly and Company. ESM, MDR, and JAJ are employees of Eli Lilly and hold stock and stock options in the company. RLO is a former employee of Eli Lilly but does not hold stock or stock options in Eli Lilly.

## Authors' contributions

MDR, RK, and LS initiated the study and developed the conceptual framework for the microsimulation model. ESM, LS, RK, RLO and JAJ finalized the design of the model and parameters to be used in the model. LS programmed the microsimulation portion of the model. RK was the lead analyst who obtained and compiled the results. ESM drafted the manuscript with substantial input from JAJ and RWK. Important intellectual contributions on the content of the manuscript were received from MDR, LS, and RLO. All authors approved the final version of the manuscript.

## Pre-publication history

The pre-publication history for this paper can be accessed here:



## Supplementary Material

Additional File 1 **Osteopenia_Mansuscript Appendix**. The appendix contains additional technical details of the methods and results of the microsimulation model. Click here for file
