# Supplementary material for: Cost-effectiveness of preventative therapies for postmenopausal women with osteopenia
Source: BMC Womens Health. 2007 Apr 17;7:6. doi: 10.1186/1472-6874-7-6 (PMC1866224; doi:10.1186/1472-6874-7-6)
Supplement: Additional File 1 — Osteopenia_Mansuscript Appendix. The appendix contains additional technical details of the methods and results of the microsimulation model. [file 1472-6874-7-6-S1.doc]

**Cost-effectiveness of Preventative Medical Therapy for Osteopenic Women: Technical Appendix**

Eric S. Meadows, PhD^1*^

Robert Klein, MS ^2^

Matthew D. Rousculp, PhD, MPH^1^

Lee Smolen, MS ^2^

Robert L. Ohsfeldt, PhD^3^

Joseph A. Johnston, MD, Msc^1^

^1^ Eli Lilly and Company, Indianapolis, IN

^2^ Medical Decision Modeling Inc., Indianapolis, IN

^3^ Texas A&M Health Science Center, College Station, TX

* Corresponding Author

Lilly Corporate Center, Mail Drop 5024

Indianapolis, Indiana 46285

[emeadows@lilly.com](mailto:ABC@lilly.com)

Tel. (317) 277-4622

Fax. (317) 277-7444

Funding provided by Eli Lilly and Company

The information included in this technical appendix is a supplement to the manuscript and summarizes additional methodological details and model outputs that could not be included in the manuscript due to space limitations. Further information is available upon request from the authors (emeadows@lilly.com).

# Additional Methodological Details

## Model Structure

**Appendix Figure 1** depicts the structure of the model and health states that were included. Events were accumulated as logical (yes/no) variables in an attribute array with dimensions equal to the number of cycles by the number of event types. Unlike a conventional Markov model, previous events affect the probabilities of future events and multiple events can occur in the same cycle.

**Appendix Figure 1.**

## Fracture Incidence

While there is much current debate about whether T-scores or a patient’s absolute risk of fracture is the better paradigm for diagnosing and managing patients with an elevated risk of fracture, (Kanis, 2002 & 2005) there are not yet widespread tools in use to calculate a patient’s absolute risk of fracture in clinical practice. Because the T-score remains the diagnostic method of choice for most clinicians who manage postmenopausal women with low bone mass, we used that approach here. Age-dependent incidences for spine (both radiographic and clinical), hip, wrist and other fractures were calculated from the National Osteoporosis Foundation's "Tabled values for 5-year risk" (NOF, 98), which is based largely on the Study of Osteoporotic Fractures. First, exponential interpolation was used between the tabled 5-year risks, and then these values were annualized. The average annual risk from the 5 years was assumed to be the 1-year risk for the 3^rd^ year of the 5 year time period. Using the NICE methodology, (NICE, 2004) we applied age-dependent multipliers to reduce whole population rates to obtain the risks for women with a Z-score of 0 and no prior fracture. Rates for fractures other than the hip, distal forearm, and spine were further reduced by 38% to eliminate toe, finger, and toe-finger-like fractures. (Seely, 1991) We assumed that 35% of spine fractures would be clinically apparent. (Schousboe, 2005; Melton, 1993) Thus, the radiographic vertebral fracture rate was set at 1.86 times the clinical vertebral fracture rate.

Post-fracture risk multipliers from Stevenson (2005) were used to calculate increased rates for all subsequent fracture. In the event a patient had experienced two or more types of fracture, the model used the highest relevant increase in subsequent fracture risks.

## Attributable Mortality from Fracture

Many studies have reported an increase in mortality following hip fracture (e.g. Cauley 2000, Johnell 2004), however, much of the increased mortality has been attributed to comorbities in the patients who fracture. For example, Kanis, Oden et al. (2003) found that 17-32% of the excess mortality rate after hip fractures was causally linked to the hip fracture event. In a previous cost-effectiveness model of raloxifene, Kanis, Borgstrom et al. (2004), based on Johnell (2004), assumed that at age 60, 23% of the excess mortality after a hip fracture was attributable to the fracture event. We used the same approach in the current model.

Johnell (2004) also found increased mortality after vertebral fractures, however an advisory panel has suggested that further research is needed on this topic.(Coyle and Tosteson, 2003) To be conservative, in the base case no increase in mortality was attributed to vertebral fractures. In sensitivity analyses, the relative mortality risks after a spine fracture was set at 2.5 in year 1, then reduced to 1.3 in years 2 through 5. (Kanis 2005) In the event of recent hip and spine fractures, the higher of the 2 different relative mortality risks was used.

## Breast Cancer Incidence

Five-year risks of invasive breast cancer were obtained from SEER (2000-2002 data) and annualized in the same manner as fracture risks. Values for stage at diagnosis were calculated by pooling 1996-2000 with 2001-2 data from the National Breast and Cervical Cancer Early Detection Program (NBCCEDP) available from the Centers for Disease Control. (CDC, 2005) Data for ages 50-59 were similar to and pooled with data for ages 60-64. A separate stage distribution for ages 65+ was calculated. Only cancers with stage information were included; we then renormalized so that cancers without stage information were assumed to be diagnosed in the same stage proportions. An alternate data source (Surveillance, Epidemiology, and End Results, SEER) indicated a somewhat higher proportion of advanced cancers, but we chose the lower rates from the CDC for two reasons. First, if breast cancer is detected at an earlier stage, the benefit of reducing the risk of developing breast cancer is lower, which would bias the model against raloxifene. Thus we felt using the CDC data was the more conservative option. Second, patients treated with raloxifene might be more likely to be screened for breast cancer and therefore might be more similar to the patients included in the NBCCEDP than the general population data reported in SEER.

Breast cancer mortalities are age dependent and computed by stage at diagnosis from stage distributions, (CDC 2005) 5-year survival rates from the SEER, (2002) and overall mortality data. (CDC 2002)

## VTE Incidence

The incidence of VTE in 67 year old women eligible for raloxifene treatment was obtained from the placebo group of the MORE trial (Grady, 2004) and extrapolated to other ages (Silverstein, 1998) as shown in **Appendix Figure 2**. An alternative age-dependent VTE function was considered. (Anderson, 1991) Silverstein was selected rather than Anderson because Silverstein included more recent data and reported a higher baseline VTE rate that would be more conservative with respect to raloxifene’s cost-effectiveness. Patients with cancer, planning to undergo surgery, or with a history of VTE were not considered appropriate patients in the current model to be consistent with the Evista prescribing information. (Eli Lilly, 2002)

**Appendix Figure 2. Age-Dependent Annual Incidences of Venous Thromboembolism in the Untreated Cohort.**

## Overall Mortality

Since deaths from breast cancer, hip fractures, and VTE are modeled explicitly, in the extraskeletal model their age-dependent proportions of overall deaths were subtracted from the background mortality. (CDC, 2002)

## Utilities

We used the same baseline no-event age-specific health state utilities for healthy postmenopausal women reported by Schousboe et al. (2005). The relative utility weights for fractures and VTE were from Kanis et al. (2004) and applied to the baseline utility multiplicatively. For example, starting with an initial utility of 0.84, a patient with a vertebral fracture (preference of 0.69) and a VTE (preference of 0.9) in the same year would have been assigned a relative utility of (0.84 * 0.69 * 0.9) = 0.52 for the 1^st^ year following these events.

Multiple sources were assimilated to assign stage-specific breast cancer utilities for the first year, years two through five, and subsequent years.(Launois, 1996; Norum, 1997; Brown, 2001; Brown 1998). Health state preferences were not available specifically by stage, so these utilities were estimated. The estimates were made such that the overall utility calculated from a weighted average of the stage-specific utilities and the stage distributions resulted in overall health state utility values for the 1^st^ and subsequent years that were consistent with those in the literature, namely 0.6 and 0.7 respectively.(Launois, 1996; Norum, 1997; Brown, 2001; Brown 1998) The relative utility of terminal breast cancer was assumed to be lower (0.23) for the year in which a patient died from breast cancer (Brown, 1998). Although limited reliable data were available for the preferences for breast cancer health states, the model results were relatively insensitive to these values. For example, varying the breast cancer disutilities by +/-30% resulted in incremental cost-effectiveness ratios that were less than 8% different from the base case (see Figure 3 in the manuscript).

# Summary of additional model inputs

**Appendix Table 1. Additional model inputs not included in Table 1 of the manuscript.**

Abbreviations: BMD = Bone mineral density, VTE = venous thromboembolism.

# Additional Results

The results below are additional model outputs that were not included in the manuscript due to space limitations.

## Base case results

The following tables summarize the incremental cost and effectiveness results with raloxifene and alendronate for a variety of ages and bone mineral density T-scores. All of these tables use the base case model parameters. Incremental cost-effectiveness ratios (ICER) are provided relative to the next-best alternative that is not dominated. Alendronate vs. placebo ICERs are also provided when alendronate is dominated.

**Appendix Table 2. Base case results varying age and T-score**

* Dominated by extended dominance (a more effective therapy with a lower incremental cost-effectiveness ratio exists)

**Appendix Table 2, continued. Base case results**

* Dominated by extended dominance (a more effective therapy with a lower incremental cost-effectiveness ratio exists)

* Dominated by extended dominance (a more effective therapy with a lower incremental cost-effectiveness ratio exists)

**Appendix Table 2, continued. Base case results**

* Dominated by extended dominance (a more effective therapy with a lower incremental cost-effectiveness ratio exists)

## Costs

The following table summarizes the costs of events included in the model. These results are for 60 year old women, T-score = -1.8 with the population mean risk of breast cancer.

**Appendix Table 3. Summary of costs for conservative care and incremental costs associated with therapy.**

All costs for raloxifene or alendronate therapy are net costs with therapy and are incremental costs or savings associated with therapy. Although the costs of treating cancer increase with later stage at diagnosis, because more cases of breast cancer are diagnosed at earlier stages, more cost savings are associated with reducing the incidence of breast cancers at earlier stages.

## Event rates with therapy

The following graph summarizes the expected number of events decreased and increased per 1,000 women treated with raloxifene at various ages, assuming a T-score of -1.8 at each age.

**Appendix Figure 3. Net events per 1,000 women treated with raloxifene.**

## Probabilistic Sensitivity Analyses (PSAs)

The following figures demonstrate the variability in the results due to uncertainty in the model parameters. The input values were sampled from lognormal distributions for event costs. The ranges shown in **Appendix Table 4** were computed assuming that the lognormals have underlying normals with standard deviations of 0.6 when the lognormal mean is < $10000, 0.5 between $10000 and $25000, and 0.4 when > $25000. Generalized beta distributions were used for four other categories of input variables: utilities, vertebral fracture risk reduction from raloxifene or alendronate, breast cancer risk reduction from raloxifene, and VTE risk increase from raloxifene. The ranges for the risks (**Appendix Table 5**) were calculated assuming the confidence intervals published from the clinical trials were the 2.5 and 97.5 percentile values. The fracture utility ranges (**Appendix Table 6**) were adapted from Schousboe (2005). Other utility ranges were assumed to have a comparable range. The beta distributions were generated assuming that the standard deviation is one-sixth of the mean.

**Appendix Table 4. Distributions for PSAs: Cost parameters**

**Appendix Table 5. Distributions for PSAs: Relative risks with treatment**

**Appendix Table 6. Distributions for PSAs: Utility parameters**

**Appendix Figure 4** illustrates the effect of including the nonskeletal risks and benefits of raloxifene. When only fracture events are considered, alendronate would be the preferred therapy compared to raloxifene at all willingness-to-pay thresholds, but neither therapy is likely to be considered cost-effective at commonly accepted societal willingness-to-pay thresholds. If the extraskeletal effects of raloxifene are included, approximately 50% of the simulations result in a cost-effectiveness ratio for raloxifene of less than $50,000/QALY compared to conservative care. Over 90% of the simulations result in cost-effectiveness ratios less than $100,000/QALY.

**Appendix Figure 4. Probabilistic sensitivity analyses including or excluding extraskeletal effects of raloxifene.**

The importance of the patient population’s age on the cost-effectiveness of raloxifene is shown in **Appendix Figure 5.** Approximately 50% of the simulations at age 55 and 60 result in cost-effectiveness ratios for raloxifene of less than $50,000/QALY compared to conservative care. Over 90% of the simulations at age 55 or 60 result in cost-effectiveness ratios less than $100,000/QALY. The proportion of the simulations that would be considered cost-effective at any willingness-to-pay threshold decrease as age increases above age 60.

**Appendix Figure 5. Probabilistic sensitivity analyses of raloxifene for various patient population ages.**

The importance of the patient population’s risk of breast cancer on the cost-effectiveness of raloxifene is shown in **Appendix Figure 6.** Using an age-dependent population average 5-year risk of breast cancer, approximately 50% of the simulations result in a cost-effectiveness ratio for raloxifene of less than $50,000/QALY. Over 85% of the simulations for a patient population with 1.5 times the age-dependent population average 5-year risk of breast cancer result in cost-effectiveness ratios less than $50,000/QALY.

**Appendix Figure 6. Probabilistic sensitivity analyses of raloxifene for patient populations with varying risk of breast cancer.**

The importance of the patient population’s bone mineral density T-score on the cost-effectiveness of raloxifene is shown in **Appendix Figure 7.** Compared to differences in the patient population risk of breast cancer (**Appendix Figure 6**), changes in the T-score within the osteopenic range had a lesser effect on the cost-effectiveness ratio.

**Appendix Figure 7. Probabilistic sensitivity analyses of raloxifene for patient populations with varying T-scores.**

#

The importance of the patient population’s bone mineral density T-score on the cost-effectiveness of alendronate is shown in **Appendix Figure 8.** Compared to raloxifene (**Appendix Figure 7**), changes in the T-score had a much greater effect on the cost-effectiveness ratio for alendronate. As T-scores worsened and approached the osteoporotic range, the proportion of the simulations that would be considered cost-effecitve at any given willingness-to-pay increased. For example, at a T-score of -1.0, essentially none of the simulations resulted in a cost-effectiveness ratio less than $100,000/QALY. For a patient population with a T-score of -2.4, nearly 90% of the simulations resulted in a cost-effectiveness ratio of less than $100,000.

**Appendix Figure 8. Probabilistic sensitivity analyses of alendronate for patient populations with varying T-scores**.

#

# Bibliography

Anderson FA Jr., Wheeler HB, Goldberg RJ, et al. A population-based perspective of the hospital incidence and case-fatality rates of deep vein thrombosis and pulmonary embolism. The Worcester DVT Study. Arch Intern Med. 1991;151:933–938.

Brown RE, Hutton J. Cost-utility model comparing docetaxel and paclitaxel in advanced breast cancer patients. Anticancer Drugs. 1998; 9:899-907.

Brown RE, Hutton J, Burrell A. Cost effectiveness of treatment options in advanced breast cancer in the UK. Pharmacoeconomics. 2001; 19:1091-102.

Cauley JA, Thompson DE, Ensrud KC, Scott JC, Black D. Risk of mortality following clinical fractures. Osteoporsis Int 2004; 11:556-61.

Cauley JA, Norton L, Lippman ME et al. Continued breast cancer risk reduction in postmenopausal women treated with raloxifene: 4-year results from the MORE trial. Breast Cancer Research and Treatment 2001; 65:125-34.

CDC. National Center for Chronic Disease Prevention and Health Promotion. National Breast and Cervical Cancer Early Detection Program. Breast Cancer Stage at Time of Diagnosis. Accessed November 2005 at <http://www.cdc.gov/cancer/nbccedp/Reports/NationalReport/tables/6.htm>

CDC, Vital Statistics of the United States: Mortality, 2002 file mortfinal2002_workipt1. Accessed December 2005 at: <http://www.cdc.gov/nchs/data/dvs/mortfinal2002_workipt1.pdf>

Coyle D, Tosteson AN. Towards a reference case for economic evaluation of osteoporosis treatments.J Rheumatol Suppl. 2003 Dec;68:31-6.

Cummings SR, Black DM, Thompson DE, Applegate WB, Barrett-Connor E, Musliner

TA, Palermo L, Prineas R, Rubin SM, Scott JC, Vogt T, Wallace R, Yates AJ,

LaCroix AZ. Effect of alendronate on risk of fracture in women with low bone density but without vertebral fractures: results from the Fracture Intervention Trial.

JAMA. 1998 Dec 23-30;280(24):2077-82.

Cundiff DK. Anticoagulation Therapy for Venous Thromboembolism. Medscape General Medicine 2004; 6(3):5. Accessed December 2005 at [http://www.medscape.com/viewarticle/487577](http://www.medscape.com/viewarticle/487577_print).

Eckman MH, Levine HJ, Salem DN, Pauker SG. Making decisions about antithrombotic therapy in heart disease: decision analytic and cost-effectiveness issues. Chest. 1998; 114(5 Suppl):699S-714S.

Gabriel SE, Tosteson AN, Leibson CL, et al. Direct medical costs attributable to osteoporotic fractures. Osteoporosis Int 2002; 13:323-30.

Gould MK, Dembitzer AD, Doyle RL, Hastie TJ, Garber AM. Low-molecular-weight heparins compared with unfractionated heparin for treatment of acute deep venous thrombosis. A meta-analysis of randomized, controlled trials. Ann Intern Med. 1999; 130:800-9.

Grady D, Ettinger B, Moscarelli E, et al. Safety and Adverse Effects Associated with Raloxifene: Multiple Outcomes of Raloxifene Evaluation. Obstetrics & Gynecology 2004; 104:837-44.

Heit JA, Mohr DN, Silverstein MD, et al. Predictors of Recurrence After Deep Vein Thrombosis and Pulmonary Embolism. Arch Intern Med 2000; 160:761-8.

Heit JA, Silverstein MD, Mohr DN, et al. Predictors of Survival After Deep Vein Thrombosis and Pulmonary Embolism. Arch Intern Med 1999; 159:445-53.

Johnell O, Kanis JA, Oden A, et al. Mortality after osteoporotic fractures. Osteoporsis Int 2004; 15:38-42.

Kanis JA, Johnell O, Black DM, et al. Effect of raloxifene on the risk of new vertebral fracture in postmenopausal women with osteopenia or osteoporosis. A reanalysis of the multiple outcomes of raloxifene evaluation trial. Bone 2003; 33:293-300.

Kanis JA, Oden A, Johnell O, et al. The components of excess mortality after hip fracture. Bone 2003; 32: 468-73.

Kanis JA, Borgstrom F, Johnell O, Jonsson B. Cost-effectiveness of risedronate for the treatment of osteoporsis and prevention of fractures in postmenopausal women. Osteoporsis Int 2004; 15:862-71.

Kanis JA, Borgstrom F, Johnell O, et al. Cost-effectiveness of raloxifene in the UK: an economic evaluation based on the MORE study. Osteoporsis Int 2005; 16:15-25.

Kanis JA, Johnell O, Oden A, et al. The risk and burden of vertebral fractures in Sweden. Osteoporosis Int 2004; 15:20-6.

Kanis JA, Black D, Cooper C et al. A new approach to the development of assessment guidelines for osteoporosis. Osteoporos Int. 2002; 13: 527-536.

Kanis JA, Borgstrom F, De Laet C et al. Assessment of fracture risk. Osteoporos Int. 2005; 16: 581-589.

Kniffin WD Jr., Baron JA, Barrett J, et al. The epidemiology of diagnosed pulmonary embolism and deep venous thrombosis in the elderly. Arch Intern Med. 1994;154:861–866

Knight KK, Wong J, Hauch O, et al. Economic and Utilization Outcomes Associated with Choice of Treatment for Venous Thromboembolism in Hospitalized Patients. Value in Health 2005; 8:191-200.

Hansson PO, Welin L, Tibblin G, et al. Deep vein thrombosis and pulmonary embolism in the general population. “The Study of Men Born in 1913.” Arch Intern Med. 1997;157:1665–1670.

Launois R, Reboul-Marty J, Henry B. A cost-utility analysis of second line chemotherapy in metastatic breast cancer: Docetaxel versus paclitaxel versus vinorelbine. Pharmacoeconomics 1996; 10:504-21.

Legorreta AP, Brooks RJ, Leibowitz AN, et al. Cost of breast cancer treatment: A 4-year longitudinal study. Arch Intern Med 1996; 156:2197-201.

Leibson CL, Tosteson AN, Gabriel SE, et al. Mortality, disability, and nursing home use for persons with and without hip fracture: a population-based study. J Am Geriat. Soc. 2002; 50:1644:50.

Melton LJ 3^rd^, Lane AW, Cooper C, et al. Prevalence and incidence of vertebral deformities. Osteporosis Int 1993; 3:113:9.

National Osteoporosis Foundation. Osteoporosis: Review of the evidence for prevention, diagnosis and treatment and cost-effectiveness analysis. Osteoporosis Int 1998;8 (Suppl 4):S1-S88.

National Institute for Clinical Excellence. The Clinical Effectiveness and Cost Effectiveness of Prevention and Treatment of Osteoporosis. 2004.

Norum J, Olsen JA, Wist EA. Lumpectomy or mastectomy? Is breast conserving surgery too expensive? Breast Cancer Res Treat 1997; 45:7-14.

Schousboe JT. Nyman JA. Kane RL. Ensrud KE. Cost-effectiveness of alendronate therapy for osteopenic postmenopausal women. Annals of Internal Medicine. 142(9):734-41, 2005 May 3.

Seeley DG, Browner WS, Nevitt MC, et al. Which fractures are associated with low appendicular bone mass in elderly women? Study of Osteoporotic Fractures Research Group. Ann Intern Med 1991; 115:837–842.

SEER 13 Registries for 2000-2002. Probability of Developing Cancer Breast Cancer by Race. Accessed November 2005 at: [http://canques.seer.cancer.gov/cgi-bin/cq_submit?dir=devcan2002&db=1&rpt=TAB&sel=1^10^^3^10^^&x=Starting%20Age^0,1,2,3,4,5,6,7,8,9,10,11,12,13,14,15,16,17,18,19&y=Ending%20Age^1,2,3,4,5,6,7,8,9,10,11,12,13,14,15,16,17,18,19,20&z=Race^1,2,3&dec=3&title=Probability%20of%20Developing%20Cancer~Breast%20Cancer%20by%20Race~SEER%2013%20Registries%20for%202000-2002](http://canques.seer.cancer.gov/cgi-bin/cq_submit?dir=devcan2002&db=1&rpt=TAB&sel=1^10^^3^10^^&x=Starting Age^0,1,2,3,4,5,6,7,8,9,10,11,12,13,14,15,16,17,18,19&y=Ending Age^1,2,3,4,5,6,7,8,9,10,11,12,13,14,15,16,17,18,19,20&z=Race^1,2,3&dec=3&title=Probability of Developing Cancer~Breast Cancer by Race~SEER 13 Registries for 2000-2002)

SEER Cancer Statistics Review 1975-2001. Female Breast Cancer (Invasive). Survival Rates by Race, Diagnosis Year, Stage and Age. Accessed November 2005 at:

[http:/seer.cancer.gov/csr/1975_2001/results_single/sect_04_table.07.pdf](http://seer.cancer.gov/csr/1975_2001/results_single/sect_04_table.07.pdf)

Stevenson M, Lloyd Jones M, De Nigris E, Brewer N, Davis S, Oakley J.

A systematic review and economic evaluation of alendronate, etidronate,

risedronate, raloxifene and teriparatide for the prevention and treatment of

postmenopausal osteoporosis. Health Technol Assess. 2005 Jun;9(22):1-160. Available online at <http://www.ncchta.org/execsumm/summ922.htm>.

White RH. The epidemiology of venous thromboembolism. Circulation 2003; 107:I4-8.
